# Occurrence and Co-Occurrence of Mycotoxins in Cereal-Based Feed and Food

**DOI:** 10.3390/microorganisms8010074

**Published:** 2020-01-03

**Authors:** Roberta Palumbo, Alfonso Crisci, Armando Venâncio, José Cortiñas Abrahantes, Jean-Lou Dorne, Paola Battilani, Piero Toscano

**Affiliations:** 1 Department of Sustainable Crop Production (DI.PRO.VE.S.), Faculty of Agriculture, Università Cattolica del Sacro Cuore, Via Emilia Parmense 84, 29100 Piacenza, Italy; roberta.palumbo@unicatt.it; 2 National Research Council, Institute of BioEconomy (IBE), 50145 Florence, Italy; alfcrisci@gmail.com (A.C.); piero.toscano@cnr.it (P.T.); 3 Centre of Biological Engineering (CEB), University of Minho, 4710-057 Braga, Portugal; avenan@deb.uminho.pt; 4 European Food Safety Authority (EFSA), 43126 Parma, Italy; jose.cortinasabrahantes@efsa.europa.eu (J.C.A.); jean-lou.dorne@efsa.europa.eu (J.-L.D.)

**Keywords:** modified mycotoxins, fumonisin, aflatoxin, deoxynivalenol, maize, wheat, oat, barley, rice, extensive literature search

## Abstract

Dietary (co)-exposure to mycotoxins is associated with human and animal health concerns as well as economic losses. This study aims to give a data-based insight from the scientific literature on the (co-)occurrence of mycotoxins (i.e., parent and modified forms) in European core cereals, and to estimate potential patterns of co-exposure in humans and animals. Mycotoxins were mainly reported in wheat and maize showing the highest concentrations of fumonisins (FBs), deoxynivalenol (DON), aflatoxins (AFs), and zearalenone (ZEN). The maximum concentrations of FB_1_+FB_2_ were reported in maize both in feed and food and were above legal maximum levels (MLs). Similar results were observed in DON-food, whose max concentrations in wheat, barley, maize, and oat exceeded the MLs. Co-occurrence was reported in 54.9% of total records, meaning that they were co-contaminated with at least two mycotoxins. In the context of parental mycotoxins, co-occurrence of DON was frequently observed with FBs in maize and ZEN in wheat; DON + NIV and DON + T2/HT2 were frequently reported in barley and oat, respectively. Apart from the occurrence of ZEN and its phase I and phase II modified forms, only a limited number of quantified data were available for other modified forms; i.e., mainly the acetyl derivatives of DON. Data gaps are highlighted together with the need for monitoring studies on multiple mycotoxins to identify co-occurrence patterns for parent mycotoxins, metabolites, and their modified forms.

## 1. Introduction

Mycotoxins are toxic secondary metabolites produced by different genera of filamentous fungi that infect susceptible plants throughout the world [1,2]. These toxins are low molecular weight and very stable compounds likely to contaminate dietary staple foods, particularly cereals, along the entire production chain, especially under conductive pre- and post-harvest conditions. Crops may be infected with multiple species of mycotoxigenic fungi, and most fungal strains produce more than one type of mycotoxin. Therefore, co-contamination of agricultural products with multiple mycotoxins is frequently observed and recently emphasized [3,4,5,6]. When raw materials are mixed to produce feed or processed into food, mycotoxin co-occurrence becomes even more likely. Although potential interventions to prevent field outbreaks have been considered in several crops worldwide [7,8,9,10,11], mycotoxins still represent an important public health and economic burden.

To date over 400 different mycotoxins have been identified with different chemical structures and properties, produced by a range of different fungal species. Among them, there are well characterized groups of mycotoxins such as aflatoxins (AFs), fumonisins (FBs), type A trichothecenes (e.g., T-2 and HT-2 toxin), type B trichothecenes (e.g., deoxynivalenol (DON), nivalenol (NIV)), zearalenone (ZEN), ochratoxin A (OTA), patulin (PAT), ergot alkaloids (EAs), as well as emerging toxins namely citrinin (CIT) and enniatins (ENNs). Of note, many structurally related congeners, defined as modified mycotoxins, are generated by plant, fungi metabolism, or food processing, and coexist with their native forms [12]. As a consequence of their complex and variable chemical structure and ubiquitous presence, humans and animals can be potentially exposed to single or multiple mycotoxins through the consumption of contaminated diets.

Mycotoxins are well established to have a number of health impacts both in humans and animals. Depending on the quantities consumed, mycotoxins and their metabolites are associated with severe acute poisoning, including death, and chronic adverse health effects. The toxicity of several mycotoxins has been demonstrated for single compounds. Aflatoxin B_1_ (AFB_1_) was classified by the International Agency for Research on Cancer (IARC) as carcinogenic to humans (Group 1), and recognized as one of the most potent liver genotoxic carcinogens [13]. Fumonisins B_1_ and B_2_ (FB_1_, FB_2_) and OTA were classified in Group 2B, compounds considered carcinogenic to animals and possibly carcinogenic to humans [13]. IARC recently also associated AFs and FBs dietary exposure with high levels of stunting and growth impairment in children.

In addition, interaction effects (i.e., additive, synergistic, or antagonistic) have also been associated with the co-exposure to multi-mycotoxin. However, in the peer-reviewed literature there are still limited papers addressing toxicokinetics (TK) aspects after concurrent exposure to mycotoxins in living organisms [14,15,16].

The effect of feed-borne mycotoxins on food-producing animal performance represents an economic problem for farmers; reduced growth, decreased egg and milk production, lower reproductive efficiency, and increased susceptibility to stress are all consequences of mycotoxin exposure. Moreover, consumers are potentially also exposed indirectly, due to the contamination in foods of animal origin due to carry-over (i.e., milk, eggs, etc.).

Multiple mycotoxins in feed and food have been recognized by European regulatory bodies as emerging risks in food safety and security with regards to animal and human health. Efforts to reduce human and animal exposure to mycotoxins resulted in the establishment of regulatory limits and monitoring programs worldwide. Maximum permitted levels (MLs) or guidance of safety levels have been provided in different countries. European legislation protects consumers by setting legal MLs for the main classes of mycotoxins in several core commodities intended for food and feed, like cereals, nuts, fruits, and derived products, including milk [17,18,19]. However, the current MLs do not consider the exposure to multiple mycotoxins and they are either based on the risk assessment of a single compound or on their sum, like the cases of AFs and FBs. According to the European Commission Regulation 1881/2006, and subsequent amendments, the MLs for AFs in cereals intended for direct human consumption is set to 2 µg/kg of AFB_1_ and 4 µg/kg of the total sum of AFB_1_, AFB_2_, AFG_1_, and AFG_2_; whereas, the MLs for the sum of FB_1_ and FB_2_ is set to 1000 µg/kg in maize intended for direct human consumption, and 4000 µg/kg in unprocessed maize [20,21]. In addition, guidance values for the sum of FB_1_ and FB_2_, and for DON have been recommended in products intended for animal feed in the EU [22].

The conventional exposure assessment paradigm of groups of populations to single mycotoxins utilizes consumption and occurrence data to derive exposure scenarios. In the context of multi-mycotoxins, a rationale way to perform risk assessments is by establishing priorities based either on the realistic frequency of the co-occurring mycotoxins or by considering the potency of the combined toxic effect.

Therefore, monitoring mycotoxin co-occurrence enables identifying the most prevalent mycotoxin mixtures and, consequently, can help to prioritize research efforts. Thus, the aim of this paper is to provide a literature and data-driven insight on the presence of mycotoxins in cereal-derived feed and food commodities in Europe, and their natural co-occurrence.

## 2. Materials and Methods

### 2.1. Data Collection and Data Extraction

**Figure 1:**
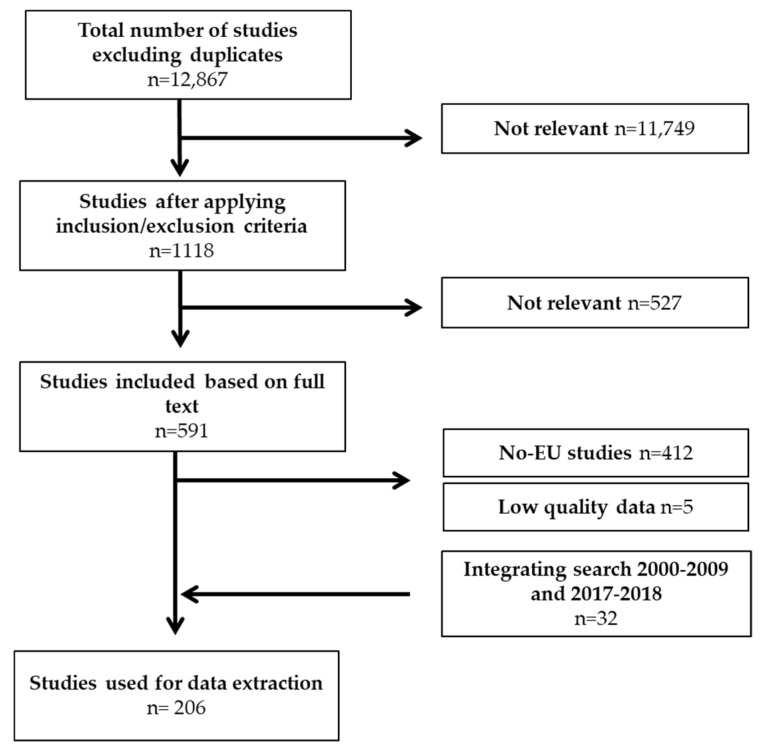
Flow chart of the extensive literature search performed.

An Extensive Literature Search (ELS) was undertaken in order to collect available papers in scientific literature on the (co-)occurrence of mycotoxins in core cereals, including maize, wheat, barley, oat, rice, rye, and sorghum from 2010 to 2018, and it was focused on the need of exposure calculations. When necessary, ad hoc searches with extended timeframes (up to 2000) were undertaken, as in the case of maize and sorghum. Mycotoxins with major public health and economic interest were included in the searching criteria, including those regulated at the European level and their modified forms, plus some emerging mycotoxins. Starting from a substantial initial number of 13,026 papers, the screening process resulted in a selection of 206 papers, which were used for data extraction. The following represents the flowchart associated with the selection of studies relevant to the aim of this study (Figure 1).

Since the collection of these data was meant to estimate dietary exposure of humans and animals in Europe, attention was paid to EU data, although the information on the origin of non-EU imported commodities was stored.

### 2.2. Development of a Structured Database on Occurrence and Co-Occurence of Mycotoxins

A database on mycotoxins occurrence/co-occurrence was structured according to the European Food Safety Authority (EFSA) Standard Sample Description version 2 (SSD2) standard [23]. The SSD2 data model was used to support reporting countries in data submissions to the EFSA and structured to collect analytical results at the sample level. In our study, the standard data model was adapted to aggregate data, which is the way authors commonly report occurrence data in the literature. However, when the co-occurrence data were reported at the sample level, a univocal identification number (ID) was assigned to each specific sample. A comprehensive description of the individual data elements of the SSD2-based data model is provided in supplementary materials.

### 2.3. Data Analysis

General qualitative and quantitative descriptions of the ELS records were conducted providing an insight of both occurrence and co-occurrence of mycotoxins in EU countries. Descriptive statistics for concentrations of the most frequently occurring mycotoxins and their modified forms in cereal- based feed and food, as well as for studies that do not specify feed or food, were derived. A qualitative score was implemented for occurrence data while frequency and multinomial distribution analysis were performed for co-occurrence data. The data model and data analysis were designed and performed in a R environment [24], respectively. All data, functions, and codes are currently available on the MYCHIF project repository [25].

### 2.4. Analysis of Occurrence Data

The database for the occurrence and co-occurrence data of mycotoxins in cereals includes 12 crop aggregations: barley, buckwheat, cereals, maize, oat, rice, rye, sorghum, spelt, triticale, wheat, and others (millet and soy). The authors noted that most often, in the case of mixed cereal grains- based commodities, the main ingredients were not indicated. For this reason, “cereals” were kept as one commodity category, intended as mixed cereals. Occurrence data for each mycotoxin, stratified by crop, were extracted and analyzed. Only records reporting concentration values (data at sample level) or mean values (aggregate data) were extracted. Values lower than the limit of detection (LOD) or lower than the limit of quantification (LOQ) were not included in the analysis, but tracked (<LOD = −1; <LOQ = −2) for further processing. Non-linear regression analysis was applied to characterize the type of distribution that best reflects each mycotoxin crop dataset block and to build a reliable reference exposure distribution that can be subsequently used for risk assessments. Weibull, gamma, lognormal, and normal distributions were tested for each data block and the benchmark with empirical data was characterized using the following:

histogram and theoretical densities plot

empirical and theoretical Cumulative Distribution Function (CDF) plot

Quantile-Quantile (Q-Q) plot

Probability-Probability (P-P) plot

To facilitate the visualization of the quality and quantity of the extracted datasets, measuring the strength of backward bibliographical context, a general scaled index based on 7 distinct scores, named Score_gen_, was implemented as the sum of 7 partial sub-indices defined below:

(1) $$\begin{array}{c} {\mathrm{Score}}_{\mathrm{gen}}={\mathrm{Score}}_{\mathrm{numerosity}}+{\mathrm{Score}}_{\mathrm{validity}}+{\mathrm{CV}}_{\mathrm{score}}+P_{\mathrm{sampleSize}}+P_{\mathrm{agePaper}}\\ +P_{\mathrm{bibIntensity}}+P_{\mathrm{haveBounds}} \end{array}$$

where Score numerosity refers to data availability (i.e., papers with at least 25 sample data were marked as 1); Score validity refers to the percentage of good data available (i.e., normalized mean of valid data given by a single paper); CV score refers to the coefficient of variation of toxin concentration calculated in records considered; P sampleSize refers to the total number of samples in all the records considered with at least 5 valid data; P agePaper refers to the age (years from the publication) of papers (i.e., normalized mean age of paper); P bibIntensity refers to bibliography intensity (i.e., normalized records of unique paper); and P haveBounds refers to records that provide also statistical information as range (i.e., Min/Max values). Each sub-index is based on data normalized in the range 0–1.

For a general view of Score_gen_ index and all sub-indices corresponding to each combination of mycotoxin and crop, heatmap plots were then produced.

### 2.5. Analysis of Co-Occurrence Data

The number of co-occurrence cases for each crop was extracted for 2 or more mycotoxins on the same sample based on the data description in each individual publication extracted from the ELS (identified as co-occurrence = 1 in the database). From all data extracted, the resulting 4 crops (maize, wheat, barley and oat), 6 main co-occurring mycotoxins and their modified forms (AFs, DON, FBs, NIV, T2+HT2, and ZEN) provided data for a more detailed analysis. Soft wheat and durum wheat were aggregated only for data analysis of co-occurrence. Finally, average concentrations and the relative frequency of co-occurrence were calculated for each crop aggregation and co-occurrence pattern.

In the context of co-occurrence of mycotoxin native forms, the frequency in which a mycotoxin was reported alone (i.e., AFs and FBs) or in combination with others was recorded, allowing the identification of patterns of co-occurrence and their frequency for each dataset. The former was used to fit a multinomial model to estimate the probability of each mycotoxin present in a food or feed sample. Estimation of such probability was performed using a multinomial model using frequencies of each combination of mycotoxin which was then simulated to estimate potential co-occurrence based on the observed patterns reported.

## 3. Results

A total number of 8406 records and 1,440,646 samples were collected. The vast majority of the studies reported data from more than one cereal, and the most studied crops were found to be wheat (34%), maize (28%), barley (10%), oat (9%), and rice (6%) (Table 1). Buckwheat, rye, triticale, sorghum, spelt, and others (millet + soy) account altogether for 7%, with rye being the most studied. Furthermore, “cereals,” accounted for 6% of total records.

**Figure 2:**
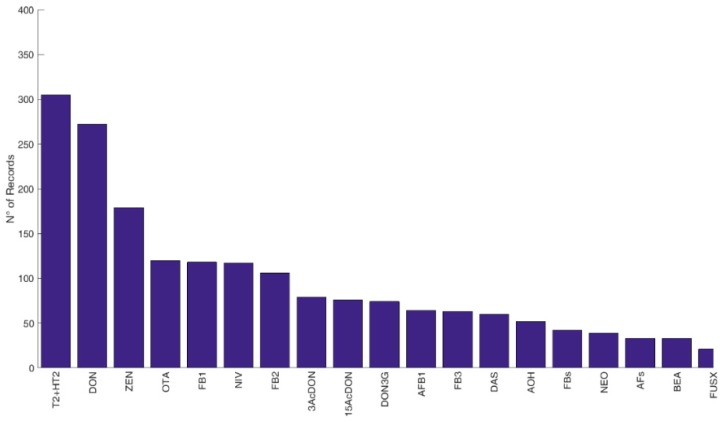
Frequencies of reported mycotoxins and secondary metabolites in feed in Europe. The figure displays the compounds with a number of records above twenty. *N* > 20: T2: T-2 toxin, HT2: HT-2 toxin, DON: deoxynivalenol, ZEN: zearalenone, OTA: ochratoxin A, FB1: fumonisin B1, NIV: nivalenol, FB2: fumonisin B2, 3Ac-DON: 3-acetyldeoxynivalenol, 15Ac-DON: 15acetyldeoxynivalenol, DON3G: deoxynivalenol-3-glucoside, AFB1: aflatoxin B1, FB3: fumonisin B3, DAS: diacetoxyscirpenol, AOH: alternariol, FBs: total fumonisins, NEO: neosolaniol, AFs: total aflatoxins, BEA: beauvericin, FUS-X: fusarenon-X. *N* < 20 (not reported in the figure): HT2-3Glc: HT-2 toxin-3-diglucoside, T2-3Glc: T-2 toxin-3-diglucoside, AFB2: aflatoxin B2, AFG1: aflatoxin G1, AFG2: aflatoxin G2, α-ZEL: α-zearalenol, FB1+FB2: fumonisin B1 + fumonisin B2, AME: alternariol monomethyl ether, STO: scirpentriol, ALTERNARIA: alternaria toxins, β-ZEL: β-zearalenol, STC: sterigmatocystin, CIT: citrinin, ENB: enniatin B, MAS: monoacetoxyscirpenol, T2-tetraol: T2 tetraol, T2-triol: T2 triol, ENA: enniatin A, ENA1: enniatin A1, ENB1: enniatin B1, ENB2: enniatin B2, ALT: altenuene, Ergocornine, Ergocristine, Ergocryptine, AND A: andrastin A, αZEL14G: α-zearalenol-14-glucoside, Marcfortine A, MON: moniliformin, NIV3G: nivalenol-3-glucoside, ROQC: Roquefortine C, β-ZEL14G: β-zearalenol-14-glucoside, TeA: tenuazonic acid, ZEN14G: zearalenone-14-glucoside, ZEN14S: zearalenone-14-sulfate, ZEN16G: zearalenone-16-glucoside.

**Figure 3:**
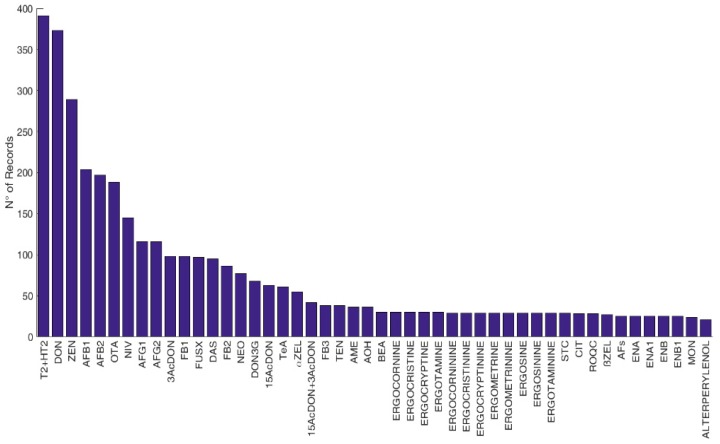
Frequency of reported mycotoxins and secondary metabolites in food in Europe. The figure displays the compounds with a number of records above twenty. *N* > 20: T2: T-2 toxin, HT2: HT-2 toxin, DON: deoxynivalenol, ZEN: zearalenone, AFB1: aflatoxin B1, AFB2: aflatoxin B2, OTA: ochratoxin A, NIV: nivalenol, AFG1: aflatoxin G1, AFG2: aflatoxin G2, 3Ac-DON: 3-acetyldeoxynivalenol, FB1: fumonisin B1, FUS-X: fusarenon-X, DAS: diacetoxyscirpenol, FB2: fumonisin B2, NEO: neosolaniol, DON3G: deoxynivalenol-3-glucoside, 15Ac-DON: 15acetyldeoxynivalenol, TeA: tenuazonic acid, α-ZEL: α-zearalenol, FB3: fumonisin B3, TEN: tentoxin, AME: alternariol monomethyl ether, AOH: alternariol, BEA: beauvericin, STC: sterigmatocystin, CIT: citrinin, ROQC: Roquefortine C, β-ZEL: β-zearalenol, AFs: total aflatoxins, ENA: enniatin A, ENA1: enniatin A1, ENB: enniatin B, ENB1: enniatin B1, MON: moniliformin. *N* < 20 (not reported in the figure): FB1+FB2: fumonisin B1 + fumonisin B2, αZEL4G: α-zearalenol-4-glucoside, βZEL4G: β-zearalenol-4-glucoside, T2-triol: T2 triol, ZEN4G: zearalenone-4-glucoside, ZEN4S: zearalenone-4-sulfate, ATX1: altertoxin 1, PAT: patulin, ATX2: Altertoxin 2, AME3G: alternariol monomethyl ether-3-glucoside, AME3S: alternariol monomethyl ether-3-sulfate, AOH3G: alternariol-3-glucoside, AOH3S: alternariol-3-sulfate, FBs: total fumonisins, AOH9G: alternariol-9-glucoside, HFB1: hydrolysed fumonisin B1, FUS: fusaproliferin, MAS: monoacetoxyscirpenol, T2-tetraol: T2 tetraol, ENB4: enniatin B4, STO: scirpentriol, αZEL14G: α-zearalenol-14-glucoside, HT2-3G: HT-2 toxin-3-diglucoside, NIV3G: nivalenol-3-glucoside, β-ZEL14G: β-zearalenol-14-glucoside, ZEN14G: zearalenone-14-glucoside, ZEN14S: zearalenone-14-sulfate, 15OHculmorin: 15-OH Culmorin, 5OHculmorin: 5-OH Culmorin, Culmorin, ENs: enniatins.

**Figure 4:**
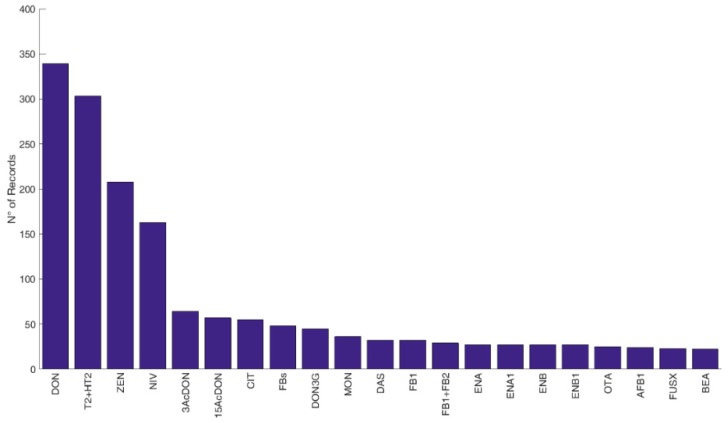
Frequency of reported mycotoxins and secondary metabolites in cereals without specifications of food or feed origin in Europe. The figure displays the compounds with a number of records above twenty. *N* > 20: DON: deoxynivalenol, T2: T-2 toxin, HT2: HT-2 toxin, ZEN: zearalenone, NIV: nivalenol, 3Ac-DON: 3-acetyldeoxynivalenol, 15Ac-DON: 15acetyldeoxynivalenol, CIT: citrinin, FBs: fumonisins, DON3G: deoxynivalenol-3-glucoside, MON: moniliformin, DAS: diacetoxyscirpenol, FB1: fumonisin B1, FB1+FB2: fumonisin B1 + fumonisin B2, ENA: enniatin A, ENA1: enniatin A1, ENB: enniatin B, ENB1: enniatin B1, OTA: ochratoxin A, AFB1: aflatoxin B1, FUS-X: fusarenon-X, BEA: beauvericin. *N* < 20 (not reported in the figure): T2-tetraol: T2 tetraol, FB2: fumonisin B2, NEO: neosolaniol, T2-triol: T2 triol, AME: alternariol monomethyl ether, AOH: alternariol, β-ZEL: β-zearalenol, STO: scirpentriol, 15Ac-DON: 15acetyldeoxynivalenol, α-ZEL: α-zearalenol, AFB2: aflatoxin B2, AFG1: aflatoxin G1, AFG2: aflatoxin G2, FB3: fumonisin B3, Culmorin: culmorin, ENB2: enniatin B2, HFB1: hydrolysed fumonisin B1, OTB: ochratoxin B, ENs: enniatins, Ergometrine/-metrinine, STC: sterigmatocystin, TeA: tenuazonic acid, TEN: tentoxin, 15OHculmorin: 15-OH Culmorin, 2-AOD-3-ol: 2-Amino-14,16-dimethyloctadecan-3-ol, Ergocryptine/-cryptinine, ATX1: altertoxin 1, Aurofusarin, Avenacein Y, Averufin, ENB3: enniatin B3, Equisetin, Ergocristine/-cristinine, ZEN4S: zearalenone-4-sulfate, Deepoxy HT2, Deepoxy T2, AFs: total aflatoxins, ALT: altenuene.

Overall, data available were classified as referring to feed (2225 records), food (4104 records), feed and food (42 records), and cereals with no defined use (2035 records). The most frequently occurring mycotoxins and modified forms (i.e., number of records above twenty) in feed, food, and cereals with no defined use are displayed in Figure 2, Figure 3 and Figure 4, respectively.

Sample origins were not always reported for European countries, even if the analyses were performed in Europe, and these included a limited number of samples originating from Africa, Asia, and South America (*n* = 590 records of which 48 records as mix from different continents), namely rice (34.2%), wheat (21.9%), maize (15.8%), sorghum (13.0%), barley (3.9%), cereals (3.7%), rye (3.6%), oat (3.1%), and soy (0.8%).

**Figure 5:**
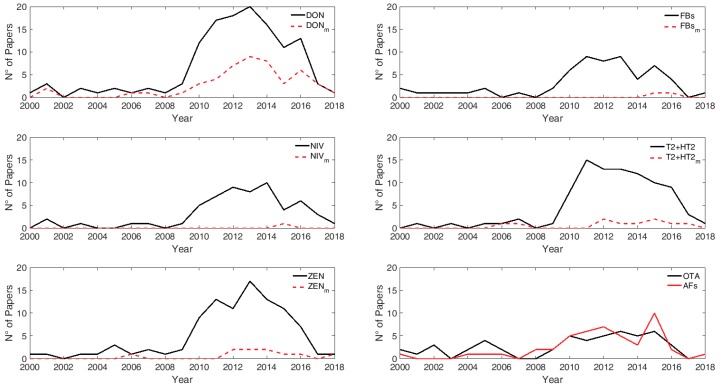
Distribution of records according to year of publication. Solid lines refer to parent mycotoxins and dashed lines refer to modified forms. m = modified forms.

Retrieved papers covered the period 2000–2018 with the majority of records distributed between 2010–2017, and the limited number of papers for the year 2018 is partly due to the limited span of the ELS for that year (i.e., last access in June 2018) (Figure 5).

The proportion of left censored data (LCD), intended as results below LOD (non-detected analytes) or below LOQ (detected but non-quantified analytes), ranged from 39.6% (<LOD) to 6.0% (<LOQ) (Table 1). Since these data were used for dietary exposure assessments in humans, these were treated by the substitution method [26,27] so that (i) at the lower-bound (LB) all results reported as lower than the LOD were set to zero and to the numerical value of the LOD for results reported as lower than the LOQ; (ii) at the upper-bound (UB), the results below the LOD were set to the numerical value of the LOD and to the value of the LOQ for results below the LOQ.

### 3.1. Data Quality

According to the data quality analysis, maize and wheat were the most studied cereals. With regards to wheat, the majority of data was reported for DON which showed the highest score with a value of 4.12/7. In maize, FB_1_ showed the highest ranking followed by DON with values of 4.08/7 and 4.06/7, respectively. Overall, DON was among the most reported mycotoxins, ranking first in wheat, barley, cereals, and rye. In maize and oat, DON ranked second after FB_1_ and T2+HT2 toxins, respectively. With regards to rice, data were reported mainly on AF and OTA with a general score ranging between 2.89 and 2.77.

**Figure 6:**
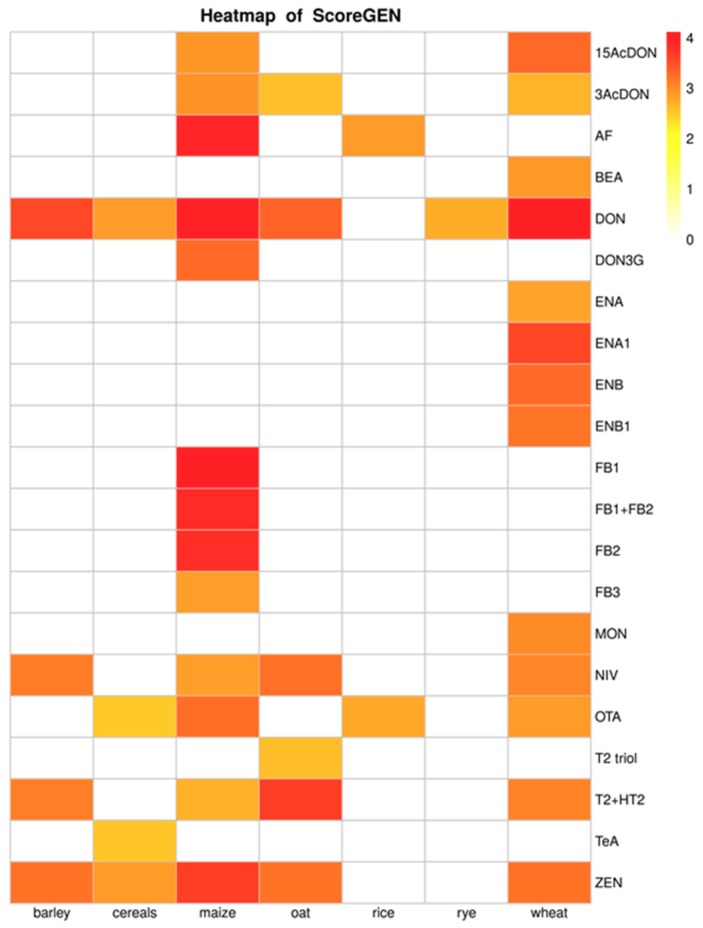
Score_gen_ heatmap for mycotoxin crops with a score higher than 1.4.

Table 2 reports the range obtained for each sub-index forming the total Score_gen_. Figure 6 provides a general view of Score_gen_ index and all sub-indices for combinations of mycotoxin and crops with a score higher than 1.4. After applying quality criteria, a final number of seven crops were selected and used for human exposure assessments to mycotoxins through cereal-based diets.

### 3.2. Occurrence of Mycotoxins

LB and UB mean concentrations, as well as maximum concentrations (UB), in food and feed are reported for each crop in the following paragraphs; more details are available in supplementary tables (Tables S1–S6), including concentrations of equivalent mycotoxin (i.e., parent and modified forms) in all cereal-based food categories at a country level in Europe (Table S7). Concentration of equivalent mycotoxins were computed and corrected on the basis of their Potency Factors (PFs) proposed by the EFSA CONTAM Panel [3,4,5].

#### 3.2.1. Wheat

Wheat was the most reported cereal with regards to individual mycotoxins (34% of total number of records). After maize, wheat contained the highest concentrations of DON reported in food (mean LB–UB: 140.1–187.9 µg/kg) and in feed, mean concentrations were reported as nearly six-fold greater (mean LB–UB: 957.7–1025.4 µg/kg). 15-Ac-DON ranged from mean concentration (LB) of 6.0 µg/kg in food and 139.1 µg/kg in feed; while 3-Ac-DON ranged from mean concentration (LB) of 8.0 µg/kg in food and 11.9 µg/kg in feed. DON3G was reported only in food (mean LB–UB: 18.1–23.6 µg/kg).

The lowest mean concentration of AFB_1_ was observed in wheat-based food (mean LB–UB: 0.0–0.6 µg/kg); however, these concentrations increased in feed (mean LB-UB: 7.4–7.6 µg/kg).

Mean concentrations (LB–UB) of ZEN ranged between 24.2–27.0 µg/kg in food and 84.6–85.7 µg/kg in feed; different modified forms were reported, with α-ZEL and β-ZEL as those with the highest mean concentrations.

Wheat was the second cereal with the highest concentration of NIV after oat (mean LB–UB: 54.8–75.2 µg/kg in food; mean LB–UB: 58.2–79.2 µg/kg in feed), and, together with barley, it was the only cereal in which NIV3G was reported.

With regard to feed, the highest concentration of OTA was reported in wheat (mean LB–UB: 12.7–13.4 µg/kg); however, in food the mean concentrations were much lower ranging between 0.5–0.8 µg/kg (LB–UB).

#### 3.2.2. Maize

Maize was the second most reported cereal after wheat with regards to individual mycotoxins and the crop contained the highest mean concentrations of FB_1_, both in food (*n* = 58; mean LB–UB: 540.7–541.3 µg/kg; max: 7878.7 µg/kg) and feed (*n* = 94; mean LB–UB: 1806.0–1807.1 µg/kg; max: 30,200.0 µg/kg). FB_2_ and FB_3_ also showed the highest mean concentrations in maize, ranging between 135.6–141.5 µg/kg and 152.6–156.2 µg/kg (LB–UB) in food and 610.7–612.2 µg/kg and 57.5–61.0 µg/kg (LB–UB) in feed, respectively. Overall, FBs were reported mainly individually, and to a lesser extent as the sum of FB_1_+FB_2_. Scarce data were reported on modified FBs (i.e., hydrolyzed FBs, HFBs) in thermally processed maize (*n* = 6; FBs+HFBs, mean: 570 µg/kg).

DON was also highly reported in maize both in food (*n* = 59; mean LB–UB: 256.3–263.2 µg/kg, max: 2266.8 µg/kg) and feed (*n* = 196; mean LB–UB: 714.9–735.6 µg/kg, max: 9528.0 µg/kg) together with its acetyl derivatives. Mean concentration of 3-Ac-DON and 15-Ac-DON in feed were respectively 26.1–27.1 µg/kg and 87.1–88.1 µg/kg (LB–UB); the lowest concentrations were reported in food for 3-Ac-DON (6.2–6.7 µg/kg), whereas 15-Ac-DON was not reported individually in food, but summed with 3-Ac-DON (mean LB–UB: 186.3–188.6 µg/kg). DON3G was also reported in maize with much higher concentrations in feed (max: 763.0 µg/kg).

AFs were also amongst the most reported mycotoxins, with AFB_1_ as the one with the highest mean concentrations (*n* = 22; mean LB–UB: 1.9–2.2 µg/kg; max: 22.4 µg/kg in food; mean: 9.9 µg/kg; max: 74.8 µg/kg in feed).

Mean concentrations of ZEN ranged between 80.6–82.1 µg/kg (LB–UB) in food and 93.3–94.9 µg/kg (LB–UB) in feed; α-ZEL and β-ZEL were the only modified forms reported in maize.

With regards to T2+HT2, low concentrations were reported in maize compared to other cereals (*n* = 53; mean LB–UB: 1.8–5.4 µg/kg); higher concentrations were reported in feed compared to food products (*n* = 174; mean LB–UB: 44.8–49.2 µg/kg). Modified forms were among the most relevant phase I metabolites, namely T2-triol and T2-tetraol, both reported in feed.

Mean concentrations (LB–UB) of NIV ranged between 9.3–28.3 µg/kg in food and 190.6–210.0 µg/kg in feed; no modified forms were reported.

Finally, mean concentrations (LB–UB) of OTA ranged between 0.3–0.6 µg/kg in food and 2.2–2.7 µg/kg in feed.

#### 3.2.3. Barley

Barley was the third most reported cereal with regards to individual mycotoxins after wheat and maize (10% of the total number of records), and showed among the highest mean concentrations for several classes of mycotoxins. With regards to food, barley showed the highest mean concentrations of ZEN (*n* = 19; mean LB–UB: 26.3–26.4 µg/kg, max: 192.0 µg/kg), OTA (*n* = 6; mean LB–UB: 1.0–1.1 µg/kg, max: 5.6 µg/kg) and T2+HT2 (*n* = 48; mean LB–UB: 27.3–30.8 µg/kg, max: 264.0 µg/kg), compared to other crops, and ranked second after maize, rice, and oat, respectively. Barley ranked third with regards to DON in food products (*n* = 22; mean: 173.8 µg/kg, max: 2029.0 µg/kg); 15-Ac-DON, 3-Ac-DON, and DON3G were also reported. In particular, the highest mean concentrations of DON3G among all cereals were reported in barley in food (*n* = 5; mean: 109.2 µg/kg, max: 390.0 µg/kg) (when LB–UB is not specified, it meant that the difference between LB and UB concentrations is not perceptible). Whereas, a low number of records was retrieved in feed (*n* = 3) with a mean DON concentration of 413.7 µg/kg; DON3G was not reported in feed. High mean concentrations were also observed for FB_1_ and FB_2_, both in food and feed; however, this information was obtained from one single record. Barley reported high concentrations of NIV in food (*n* = 16; mean LB–UB: 35.2–40.2 µg/kg), ranking third after oat and wheat; NIV3G was reported in one record (25.2 µg/kg). Information on NIV in feed were not retrieved.

#### 3.2.4. Oat

The highest concentrations of NIV were reported in oat, both in food (*n* = 3; mean LB–UB: 81.4–86.3 µg/kg) and feed (mean LB–UB: 263.3–280.0 µg/kg). FB_1_ and FB_2_ were reported only in two records respectively, one in food (FB_1_: 0.1 µg/kg; FB_2_: 0.5 µg/kg) and one in feed (FB_1_: 30.0 µg/kg; FB_2_: 28.0 µg/kg). DON ranked first among other cereals in feed (*n* = 6; mean: 1309.7 µg/kg, max: 2690.0 µg/kg), and it was reported also in food with much lower concentrations (*n* = 31; mean LB–UB: 130.6–132.6 µg/kg, max: 1230.0 µg/kg). Modified forms of DON were also reported; mean concentrations of 3-Ac-DON were higher than 15-Ac-DON both in food (mean LB–UB: 28.5–30.6 µg/kg; mean LB–UB: 6.6–10.8 µg/kg) and feed (mean LB–UB: 127.0–139.5 µg/kg; mean LB–UB: 24.5–49.5 µg/kg). DON3G showed high concentrations in feed (*n* = 2; mean: 711.0 µg/kg). Scarce information was retrieved on AFs both in food and feed; AFB_1_, AFB_2_, AFG_1_, and AFG_2_ were reported in food only in two records, whereas in feed only one record reported AFB_1_. It should be noted that the highest concentrations of T2+HT2 were reported in oat both in food (*n* = 65; mean LB–UB: 179.9–182.5 µg/kg) and feed (*n* = 17; mean LB–UB: 88.1–96.9 µg/kg).

#### 3.2.5. Rice

The majority of data for individual mycotoxins in rice regarded food commodities where the highest mean concentrations of AFB_1_ (*n* = 124; mean LB–UB: 3.1–3.3 µg/kg; max: 91.7 µg/kg) and OTA (*n* = 44; mean: 2 µg/kg in food) were reported. Low mean concentrations of FB_1_ (*n* = 3; mean LB–UB: 0.0–8.4 µg/kg; max: 12.5 µg/kg), FB2 (*n* = 1; mean LB–UB: 0.0–0.5 µg/kg; max: 0.5 µg/kg), DON (*n* = 22; mean LB–UB: 7.9–15.6 µg/kg; max: 96.0 µg/kg), T2+HT2 (*n* = 14; mean LB–UB: 0.0–8.9 µg/kg; max: 60.0 µg/kg), and ZEN (*n* = 7; mean LB–UB: 0.0–6.6 µg/kg; max: 10.1 µg/kg) were reported. No information was retrieved on modified forms in rice except for 3-Ac-DON reported in four records with mean ranging (LB–UB) between 0.0 and 0.6 µg/kg. Five records were also reported on NIV (mean LB–UB: 0.0–16.0 µg/kg; max 75.0 µg/kg). In feed, only two mycotoxins were reported, namely DON and T2+HT2.

#### 3.2.6. Rye

Overall, scarce information was available on rye compared to other cereals; the number of records ranged between one and 18, and the majority of the data retrieved was for food commodities. It could be emphasized that rye showed the highest mean concentration of OTA (mean LB–UB: 0.8–0.9 µg/kg). However, this information was derived from a limited number of records (*n* = 5). DON was reported both in food (*n* = 11; mean LB–UB: 55.9–56.8 µg/kg) and feed (*n* = 2; mean: 56.2 µg/kg). Whereas 15-Ac-DON (*n* = 2; mean LB–UB: 0.5–3.0 µg/kg) and 3-Ac-DON (*n* = 5; mean LB–UB: 8.6–13.6 µg/kg) were reported only in food.

### 3.3. Co-Occurrence of Mycotoxins

The main co-occurring mycotoxins were analyzed by crop category. The analysis of the data quality led to the identification of five suitable crop categories, namely maize, wheat, oat, barley, and cereals. The latter was often reported even if the composition and/or the percentages of ingredients were not always indicated by the authors. However, considering that the consumption of mixed cereal grains-based commodities is also one of the causes of the natural co-occurrence of mycotoxins both in animal and human diets, this information was kept.

Several surveys reported the natural co-occurrence of mycotoxins, and most of them concerned DON, OTA, NIV, ZEN, and T2+HT2. Less data were found for AFs, ENs, and *Alternaria* toxins.

**Figure 7:**
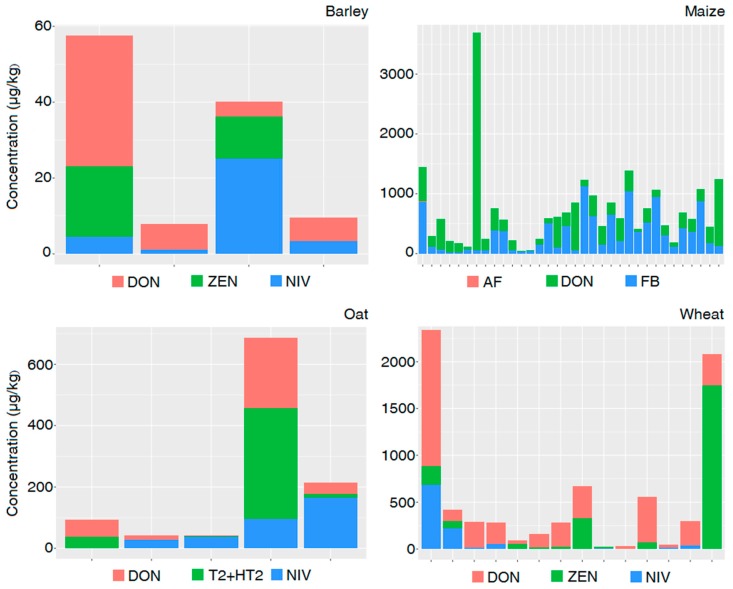
Concentrations of each co-occurring mycotoxin for barley, maize, oat, and wheat.

For each crop aggregation and co-occurrence, average concentrations were then calculated (Figure 7) In detail, for each paper reporting on co-occurrence for barley, maize, oat, and wheat, the concentration of each co-occurring mycotoxin is reported as the mean value.

### 3.4. Results of Multinomial Analysis

**Figure 8:**
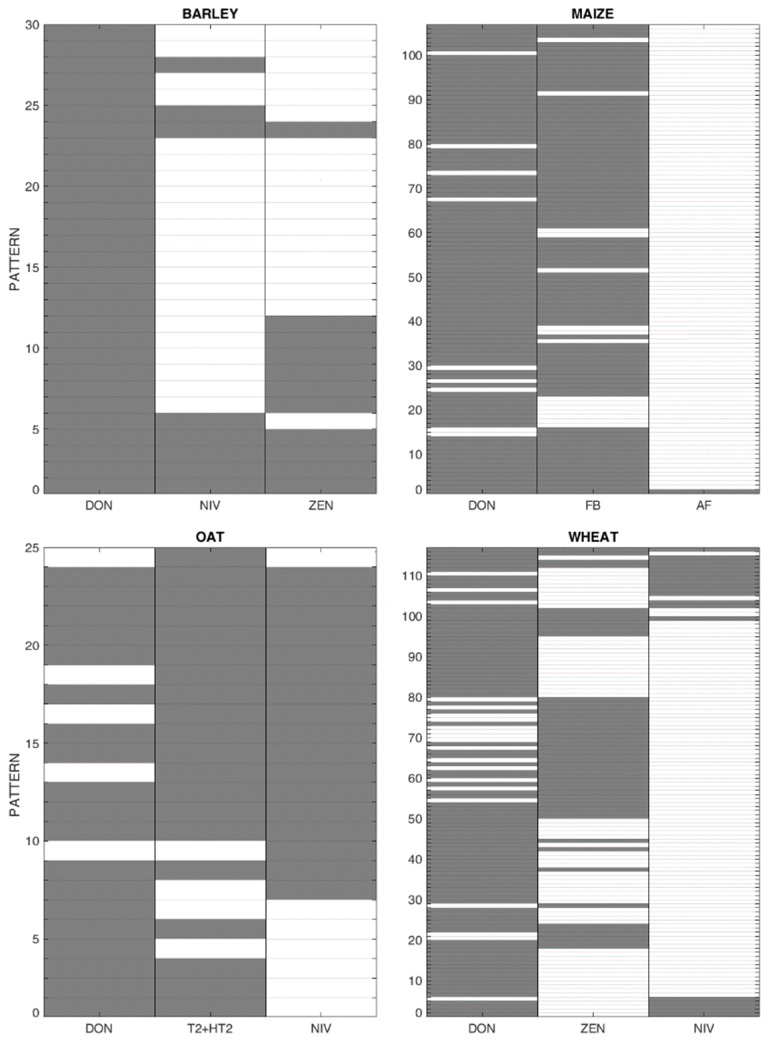
Total number of observations with specific patterns of mycotoxin co-occurrence. Grey and white boxes display the presence and absence of mycotoxins, respectively.

The multinomial analysis provided a simulation model that allowed prediction of potential co-occurrence patterns for two or more mycotoxins based on the observed patterns reported in the literature. Probabilities of mycotoxin co-occurrence for one or more mycotoxins were simulated for records above the LOD and are reported below. Figure 8 shows the number and type of observed patterns of co-occurrence of native mycotoxins in barley, maize, oat, and wheat, while the probabilities simulated by the multinomial model are reported in Table 3. In maize, DON and FB have the highest probability of co-occurrence (74.4%), whereas the probability of DON, FB, and AF is rather low (1.0%). In barley and wheat, the combination of DON and ZEN is the most probable; whereas DON and T2+HT2 have the highest simulated probability of co-occurring in oat.

## 4. Discussion

Cereals are often contaminated with a wide range of mycotoxins and other fungal metabolites. Unsurprisingly, wheat and maize were the most reported cereals with the highest concentrations of FBs, DON, AFs, and ZEN.

FBs were widely reported in maize foods and feed for which the maximum concentrations of FB_1_+FB_2_ exceeded the legal maximum levels (MLs) of 1000 and 4000 μg/kg, respectively [28].

In the context of food, the max concentrations of DON in barley, maize, oat, and wheat exceeded the legal limits of 750 μg/kg [20,28]; however, when looking at mean concentrations, none of the cereals showed very high concentrations. Similar results were observed in feed except that max concentrations in barley did not exceed the MLs of 1250 μg/kg in contrast to maize, oat, and wheat [20,28].

In line with pre-existing knowledge, maximum concentrations of T2+HT2 were particularly high in oat and oat-containing foods, exceeding the MLs of 200 μg/kg [18].

AFs were predominantly reported in rice and maize as a result of a pre- and post-harvest colonization of the grains with *A. flavus* [7]. In addition, in rice, high concentration of OTA was also reported in food, exceeding the legal limits of 3.0 μg/kg [20]. These results are in agreement with the well-known rice contamination with the OTA-producer *Aspergillus ochraceus*.

Contamination with NIV was more relevant for oat, wheat, and barley, however, MLs have not been set in the current regulation for either NIV nor for its metabolites [20].

With regards to occurrence of native forms, DON, FBs, and ZEN showed the highest simulated potential co-occurrence value, and in particular, DON was more probable to be found in co-occurrence with FBs in maize and with ZEN in wheat. This finding is consistent with the results of a recent study conducted on Canadian cereal samples where the co-occurrence of DON and other *Fusarium* mycotoxins was frequently observed in wheat and barley [29].

Overall, the data collection exercise concludes that occurrence of modified forms are mostly reported in food compared to feed. Apart from the occurrence of ZEN and its phase I and phase II modified forms, only a limited number of quantitative data are available for other modified forms; i.e., acetyl derivatives of DON, hydrolyzed FBs, phase I metabolites of T2, and NIV3G. In addition, data are still scarcely and unevenly reported regardless of an increased awareness of the contribution of modified forms to the toxicity of mycotoxins. Liquid chromatography (LC) coupled with mass spectrometry (MS) has only recently become widely used for the determination of multiple mycotoxins which partly explain why literature data are still scarce on the co-occurrence of modified forms [30]. In general, promising progresses have been recently observed in the context of analytical methods, providing a positive indication of forthcoming improvements for the simultaneous determination of multiple mycotoxins, both of different native toxins and modified forms. Yet, analytical methods are still a limiting factor for a complete data collection, both for the cost and the lack of suitable protocols.

In summary, the large body of evidence collected in this study highlights that wheat and maize may contribute significantly to mycotoxin co-exposure in human and animal species compared to other crops. The results indicate that mycotoxin co-occurrence is common in European cereal-based feed and food, and further highlights the need to conduct monitoring studies for multiple mycotoxins. Such studies would also support filling considerable data gaps regarding the co-occurrence of mycotoxins and their modified forms. Further research efforts are needed to identify co-occurrence patterns of multiple mycotoxins in the real world and these will allow provision of a scientific basis to understand the combined toxicity of mycotoxins, the relative contribution of the parent compounds compared to metabolites, and modified forms and their likely interactions.

## 5. Conclusions

Cereals and related processed food products are frequently contaminated with mycotoxins, and co-occurrence of *Fusarium* mycotoxins is highly reported in cereals of major consumption in human and animal species, particularly wheat, maize, barley, and oat. However, there is still limited knowledge on the presence and co-occurrence of multiple mycotoxins, both for native mycotoxins and their modified forms, in food and feed. Therefore, the challenge of depicting realistic patterns of co-exposure in humans and animals remains. To bring forward the risk assessment of mycotoxin mixture, the refinement of assessment factors to determine safe levels of exposure is needed, and the following is recommended:

(1) The necessity of continuous monitoring of the major mycotoxins in different agricultural commodities and the creation of harmonized methods for generating accurate (co-)occurrence data is strongly suggested. This is mandatory to provide consistent and coherent data for mycotoxin co-occurrence and will allow risk modelling to prioritize key congeners of human and animal health relevance;

(2) LODs and LOQs for mycotoxins and the analytical method used may vary significantly across studies and across measurements. It is known that the degree of LCD in the dataset has a large impact on the uncertainty of the exposure assessment; this uncertainty is further magnified when assessing exposure to multiple chemical substances. Thus, a more harmonized approach should be adopted to reduce this source of uncertainty but also to allow the usability of published data that, currently, in some cases are unusable (e.g., authors reporting a range of LOD/LOQ across different classes of mycotoxins);

(3) More accurate reporting of geographical information of the samples could also optimize the efforts to better understand and map the mycotoxin problem in the EU.

In this context, this article provides a source of ready-to-use data for the implementation of exposure assessments of multiple mycotoxins in food and feed.

## Figures and Tables

**Table 1:** Total number of records per crop with specification on the number of records below the limit of detection, the limit of quantification, and co-occurrence studies.

Crop/Aggregation	N of Records ^1^	<LOD ^2^	<LOQ ^3^	N of Co-Occ Studies ^4^	N of Co-Occ Records ^5^
Barley	865	140	109	17	330
Buckwheat	6	3	0	1	4
Cereals	463	189	61	12	223
Maize	2362	1055	66	27	1443
Oat	740	150	81	14	374
Rice	520	297	26	8	343
Rye	236	75	14	10	111
Sorghum	101	62	9	2	51
Spelt	83	26	1	3	61
Triticale	127	48	0	3	13
Wheat	2860	1252	142	43	1646
Others ^6^	43	32	0	3	13
All	8406	3329	509	482	4612

^1^ Total number of records; ^2^ Records reported as below the limit of detection; ^3^ Records reported as below the limit of quantification; ^4^ Number of co-occurrence studies; ^5^ Number of co-occurrence records; ^6^ Millet and soy.

**Table 2:** Composition of the Score_gen_ index and range for each individual sub-index

N	Sub-Indices Code	Sub-Indices Meaning	Range	Normalization
1	Score numerosity	data availability	6–332	0–1
2	Score validity	percentage of good data available	0–100	0–1
3	CV score	coefficient of variation of toxin concentration	0–1	0–1
4	P sampleSize	total samples number	1–48	0–1
5	P agePaper	age of papers	2001–2018	0–1
6	P bibIntensity	bibliography intensity	1–215	0–1
7	P haveBounds	statistical information	0–1	0–1

**Table 3:** Probability simulated by the multinomial model of having co-occurring mycotoxins in maize, barley, oat, and wheat.

**Pattern**	**DON**	**NIV**	**ZEN**	**%**	**Pattern**	**DON**	**FB**	**AF**	**%**
**Barley**					**Maize**				
1			1	1.3	1		1		10.7
2		1		0.8	2		1	1	0.5
3		1	1	4.5	3	1			13.1
4	1			20.5	4	1		1	0.3
5	1		1	32.9	5	1	1		74.4
6	1	1		25.8	6	1	1	1	1.0
7	1	1	1	14.2					
**Pattern**	**DON**	**T2/HT2**	**NIV**	**%**	**Pattern**	**DON**	**NIV**	**ZEN**	**%**
**Oat**					**Wheat**				
1			1	3.0	1			1	2.7
2		1		5.0	2		1		0.2
3		1	1	22.3	3		1	1	5.0
4	1			3.0	4	1			18.1
5	1		1	18.8	5	1		1	46.1
6	1	1		25.4	6	1	1		15.0
7	1	1	1	22.5	7	1	1	1	12.9

## Supplementary Materials

The following are available online at https://www.mdpi.com/2076-2607/8/1/74/s1.
