# Supplementary material for: Occurrence and Co-Occurrence of Mycotoxins in Cereal-Based Feed and Food
Source: Microorganisms. 2020 Jan 3;8(1):74. doi: 10.3390/microorganisms8010074 (PMC7023405; doi:10.3390/microorganisms8010074)
Supplement: Supplementary file 1 [file microorganisms-08-00074-s001.pdf]

Table S1. Occurrence and co-occurrence of DON and secondary metabolites (µg/kg) for Barley, Cereals, Maize, Oat, Rice, Rye and Wheat for feed and food products.

			**FOOD**						**FEED**					
			**15AcDON**	**15+3AcDON**	**3AcDON**	**AcDONs**	**DON**	**DON3G**	**15AcDON**	**15+3AcDON**	**3AcDON**	**AcDONs**	**DON**	**DON3G**
**Barley**	N		5	4	6	1	22	5	1		1		3	
	**Mean**	**LB**	19.6	0.3	22.3	0.0	173.8	109.2	0.0		0.0		413.7	
	**Conc**	**UB**	21.6	1.0	26.7	0.0	173.8	109.2	50.0		50.0		413.7	
	**Max Conc**	**UB**	97.0	1.0	120.0	0.0	2029.0	390.0	50.0		50.0		600.0	
**Cereals**	N		17		24		21	6					2	
	**Mean**	**LB**	9.3		14.3		46.9	22.8					543.0	
	**Conc**	**UB**	13.1		17.4		50.1	24.2					543.0	
	**Max Conc**	**UB**	119.0		130.0		132.1	29.0					884.0	
**Maize**	N			15	5		59	15	51		51	2	196	72
	**Mean**	**LB**		186.3	6.2		256.3	0.0	87.1		26.1	108.0	714.9	112.1
	**Conc**	**UB**		188.6	6.7		263.2	5.3	88.1		27.1	110.5	735.6	117.0
	**Max Conc**	**UB**		808.1	31.0		2266.8	5.3	1047.0		339.0	211.0	9528.0	763.0
**Oat**	N		21		24		31	6	2		4		6	2
	**Mean**	**LB**	6.6		28.5		130.6	34.2	24.5		127.0		1309.7	711.0
	**Conc**	**UB**	10.8		30.6		132.6	36.8	49.5		139.5		1309.7	711.0
	**Max Conc**	**UB**	27.0		116.0		1230.0	97.0	50.0		341.0		2690.0	806.0
**Rice**	N				4		22						1	
	**Mean**	**LB**			0.0		7.9						800.0	
	**Conc**	**UB**			0.6		15.6						800.0	
	**Max Conc**	**UB**			0.6		96.0						800.0	
**Rye**	N		2		5		11						2	

	**Mean**	**LB**	0.5		8.6		55.9						56.2	
	**Conc**	**UB**	3.0		13.6		56.8						56.2	
	**Max Conc**	**UB**	5.0		43.2		277.0						83.1	
**Wheat**	N		16	23	22		162	33	19		19	1	41	
	**Mean**	**LB**	6.0	2.8	8.0		140.1	18.1	139.1		11.9	16.0	957.7	
	**Conc**	**UB**	55.9	7.5	14.6		187.9	23.6	142.6		16.4	16.0	1025.4	
	**Max Conc**	**UB**	150.0	64.8	59.0		1657.0	250.0	1575.0		93.8	16.0	12270.0	

LB: lower-bound scenario where the concentration of non-detected analyte is zero and the concentration of detected but non-quantified analyte is the limit of detection. UB: upper-bound scenario where the concentration of non-detected analyte is the limit of detection and the concentration of detected but non-quantified analyte is the limit of quantification. Max Conc refers to maximum upper bound concentration value. Blanck cells refer to not available information. N: number of records.

Table S2. Occurrence and co-occurrence of FB and secondary metabolites (μg/kg) for Barley, Cereals, Maize, Oat, Rice, Rye and Wheat for feed and food products.

			**FOOD**						**FEED**					
			**FB_1**	**FB_1+FB_2**	**FB_2**	**FB_3**	**FBs**	**FBs+HFBs**	**FB_1**	**FB_1+FB_2**	**FB_1+FB_2+FB_3**	**FB_2**	**FB_3**	**FBs**
**Barley**	N		1	1	1		1		1			1		
	**Mean**	**LB**	156.3	0.0	65.0		0.0		0.0			0.0		
	**Conc**	**UB**	156.3	100.0	65.0		25.0		30.0			30.0		
	**Max Conc**	**UB**	156.3	100.0	65.0		25.0		30.0			30.0		
**Cereals**	N		5	1	4	1								
	**Mean**	**LB**	8.9	0.0	19.3	0.0								
	**Conc**	**UB**	9.9	100.0	20.5	5.0								
	**Max Conc**	**UB**	35.0	100.0	75.0	5.0								
**Maize**	N		58	13	54	23	7	6	94	13	5	85	45	37
		**LB**	540.7	823.8	135.6	152.6	472.8	570.0	1806.0	2611.8	7220.0	610.7	57.5	681.8

	**Mean Conc**	**UB**	541.3	823.8	141.5	156.2	473.7	570.0	1807.1	2611.8	7220.0	612.2	61.0	795.8
	**Max Conc**	**UB**	7878.7	4092.0	1563.6	1066.1	1300.5	1651.0	30200.0	7890.0	11100.0	13200.0	246.0	5727.0
**Oat**	**N**		1	1	1				1			1		
	**Mean**	**LB**	0.0	0.0	0.0				0.0			28.0		
	**Conc**	**UB**	0.1	100.0	0.5				30.0			28.0		
	**Max Conc**	**UB**	0.1	100.0	0.5				30.0			28.0		
**Rice**	**N**		3		1									
	**Mean**	**LB**	0.0		0.0									
	**Conc**	**UB**	8.4		0.5									
	**Max Conc**	**UB**	12.5		0.5									
**Rye**	**N**			1			1							
	**Mean**	**LB**		0.0			6.2							
	**Conc**	**UB**		100.0			6.2							
	**Max Conc**	**UB**		100.0			6.2							
**Wheat**	**N**		17	1	17	14	1		17			17	16	2
	**Mean**	**LB**	8.1	0.0	2.5	0.0	0.0		4.6			1.6	0.0	551.25
	**Conc**	**UB**	10.1	100.0	3.8	0.7	25.0		13.8			9.4	7.7	667.25
	**Max Conc**	**UB**	131.2	100.0	35.9	6.0	25.0		78.0			30.0	8.4	1102.5

LB: lower-bound scenario where the concentration of non-detected analyte is zero and the concentration of detected but non-quantified analyte is the limit of detection. UB: upper-bound scenario where the concentration of non-detected analyte is the limit of detection and the concentration of detected but non-quantified analyte is the limit of quantification. Max Conc refers to maximum upper bound concentration value.

Table S3. Occurrence and co-occurrence of AF and secondary metabolites (µg/kg) for Barley, Cereals, Maize, Oat, Rice, Rye and Wheat for feed and food products.

			FOOD					FEED				
			**AFB_1**	**AFB_2**	**AFG_1**	**AFG_2**	**AFs**	**AFB_1**	**AFB_2**	**AFG_1**	**AFG_2**	**AFs**
**Barley**	**N**		3	1	1	1	5	1				
	**Mean Conc**	**LB**	0.2	0	0.1	0	0	0				
		**UB**	0.2	0	0.1	0	0.4	0.2				
	**Max Conc**	**UB**	0.4	0	0.1	0	1.8	0.2				
**Cereals**	**N**		13	14	14	13	1					
	**Mean Conc**	**LB**	0	0	0	0	0					
		**UB**	0.6	0.9	1.1	0.2	0.4					
	**Max Conc**	**UB**	3	10	5	0.4	0.4					
**Maize**	**N**		22	22	22	22	3	35	6	6	6	27
	**Mean Conc**	**LB**	1.9	0.1	0	0	3.6	9.9	1.3	2.8	1.1	4.2
		**UB**	2.2	0.8	0.4	0.3	3.7	9.9	1.3	2.8	1.1	5.5
	**Max Conc**	**UB**	22.4	10	5	1	10.3	74.8	3.2	14	3.2	67
**Oat**	**N**		2	2	2	2	2	1				
	**Mean Conc**	**LB**	0	0.8	0	0	0	0				
		**UB**	1.6	0.9	2.8	0.2	0.4	0.2				
	**Max Conc**	**UB**	3	1.6	5	0.3	0.4	0.2				
**Rice**	**N**		124	120	35	35	5					
	**Mean Conc**	**LB**	3.1	0.2	10.7	7.8	1.4					
		**UB**	3.3	0.5	10.9	7.8	1.5					
	**Max Conc**	**UB**	91.7	12.1	78.7	31	1.9					

**Rye**	**N**		1	1	1	1	2					
	**Mean Conc**	**LB**	0	0	0	0	0					
		**UB**	0.9	0.2	2.2	0.4	1.1					
	**Max Conc**	**UB**	0.9	0.2	2.2	0.4	1.8					
**Wheat**	**N**		34	33	33	33	4	24	9	9	9	
	**Mean Conc**	**LB**	0	0	0.2	0	0.7	7.4	0	0	0	
		**UB**	0.6	0.1	1.1	0.3	0.9	7.6	0.2	0.2	0.2	
	**Max Conc**	**UB**	3	0.2	6.6	0.9	2.6	143.6	0.3	0.3	0.3	

LB: lower-bound scenario where the concentration of non-detected analyte is zero and the concentration of detected but non-quantified analyte is the limit of detection. UB: upper-bound scenario where the concentration of non-detected analyte is the limit of detection and the concentration of detected but non-quantified analyte is the limit of quantification. Max Conc refers to maximum upper bound concentration value.

Table S4 a. Occurrence and co-occurrence of ZEN and secondary metabolites (µg/kg) for Barley, Cereals, Maize, Oat, Rice, Rye and Wheat for food products.

			**FOOD**											
			**αZEL**	**αZEL14G**	**αZEL4G**	**ßZEL**	**ßZEL14G**	**ßZEL4G**	**ZEN**	**ZEN14G**	**ZEN14S**	**ZEN16G**	**ZEN4G**	**ZEN4S**
**Barley**	**N**		3	1		3	1		19	1	1	1		
	**Mean**	**LB**	0.2	2.9		0.7	0.7		26.3	2.7	10.6	0.3		
	**Conc**	**UB**	9.9	2.9		11.7	0.7		26.4	2.7	10.6	0.9		
	**Max Conc**	**UB**	27.0	2.9		31.0	0.7		192.0	2.7	10.6	0.9		
**Cereals**	**N**		6		5	6		5	18				5	6
	**Mean**	**LB**	32.7		0.0	24.7		0.0	10.6				9.6	4
	**Conc**	**UB**	34.7		9.0	28.5		9.0	11.5				14.4	13.2
	**Max Conc**	**UB**	110.0		9.0	86.0		9.0	53.0				20.0	24
**Mai ze**	**N**		15						37					

	**Mean**	**LB**	0.0						80.6					
	**Conc**	**UB**	2.5						82.1					
	**Max Conc**	**UB**	2.5						823.0					
**Oat**	**N**		8		7	8		7	26				7	6
	**Mean**	**LB**	16.1		0.0	19.5		2.9	11.4				3.4	2.0
	**Conc**	**UB**	19.5		9.0	24.1		10.6	13.0				11.4	11.2
	**Max Conc**	**UB**	68.0		9.0	96.0		20.0	85.0				16.0	12.0
**Rice**	**N**								7					
	**Mean**	**LB**							0					
	**Conc**	**UB**							6.6					
	**Max Conc**	**UB**							10.1					
**Rye**	**N**		1			1			7					
	**Mean**	**LB**	0.0			0.0			7.0					
	**Conc**	**UB**	2.0			2.0			7.3					
	**Max Conc**	**UB**	2.0			2.0			41.0					
**Wheat**	**N**		22	1	6	9	1	6	165	1	1	1	6	6
	**Mean**	**LB**	3.2	3.1	0.0	18.2	0.0	0.0	24.2	0.6	4.9	2.1	2.7	3.7
	**Conc**	**UB**	5.7	3.1	9.0	22.3	0.2	9.0	27.0	0.6	4.9	2.1	10.7	11.0
	**Max Conc**	**UB**	39.0	3.1	9.0	104.0	0.2	9.0	856.0	0.6	4.9	2.1	16.0	11.0

LB: lower-bound scenario where the concentration of non-detected analyte is zero and the concentration of detected but non-quantified analyte is the limit of detection. UB: upper-bound scenario where the concentration of non-detected analyte is the limit of detection and the concentration of detected but non-quantified analyte is the limit of quantification. Max Conc refers to maximum upper bound concentration value.

Table S4 b. Occurrence and co-occurrence of ZEN and secondary metabolites (µg/kg) for Barley, Cereals, Maize, Oat, Rice, Rye and Wheat for feed products.

			**FEED**											
			**αZEL**	**αZEL14G**	**αZEL4G**	**ßZEL**	**ßZEL14G**	**ßZEL4G**	**ZEN**	**ZEN14G**	**ZEN14S**	**ZEN16G**	**ZEN4G**	**ZEN4S**
Barley	N								3					
	Mean	LB							16.3					
	Conc	UB							16.3					
	Max Conc	UB							27.0					
Cereals	N								5					
	Mean	LB							79.9					
	Conc	UB							79.9					
	Max Conc	UB							134.0					
Maize	N		2			2			122					
	Mean	LB	9.0			91.5			93.3					
	Conc	UB	9.0			91.5			94.9					
	Max Conc	UB	15.0			166.0			2180.0					
Oat	N		2	1		2	1		5	1	1	1		
	Mean	LB	69.0	0.0		1.5	0.0		44.2	0.1	31.6	4.2		
	Conc	UB	69.0	0.5		17.0	0.2		44.2	0.3	31.6	4.2		
	Max Conc	UB	136.0	0.5		31.0	0.2		77.0	0.3	31.6	4.2		
Rice	N													
	Mean	LB												
	Conc	UB												
	Max Conc	UB												
Rye	N													

	Mean	LB													
	Conc	UB													
	Max Conc	UB													
Wheat	N		7						24						
	Mean	LB	3.5						84.6						
	Conc	UB	4.1						85.7						
	Max Conc	UB	10.0						555.0						

LB: lower-bound scenario where the concentration of non-detected analyte is zero and the concentration of detected but non-quantified analyte is the limit of detection. UB: upper-bound scenario where the concentration of non-detected analyte is the limit of detection and the concentration of detected but non-quantified analyte is the limit of quantification. Max Conc refers to maximum upper bound concentration value.

Table S5. Occurrence and co-occurrence of T2-HT2 and secondary metabolites (µg/kg) for Barley, Cereals, Maize, Oat, Rice, Rye and Wheat for feed and food products.

			**FOOD**						**FEED**					
			**T2 tetraol**	**T2 triol**	**T2G**	**T2+HT2**	**HT23G**	**HT2G**	**T2 tetraol**	**T2 triol**	**T2G**	**T2+HT2**	**HT23G**	**HT2G**
**Barley**	**N**		2	2		48	1				18	45		18
	**Mean Conc**	**LB**	51.4	10.3		27.3	3.6				2.4	53.3		48.2
		**UB**	51.4	10.3		30.8	10.8				2.4	55.6		48.2
	**Max Conc**	**UB**	102.7	20.4		264.0	10.8				14.5	213		162.8
**Cereals**	**N**					58						13		
	**Mean Conc**	**LB**				2.8						27.7		
		**UB**				9.7						27.8		
	**Max Conc**	**UB**				60.0						65.1		

**Maize**	**N**					53			3	2		174		
	**Mean Conc**	**LB**				1.8			117.7	42		44.8		
		**UB**				5.4			117.7	42		49.2		
	**Max Conc**	**UB**				60.0			301	76		2300		
**Oat**	**N**		1	13		65			1	1		17	1	
	**Mean Conc**	**LB**	3.6	20.3		179.9			150	19		88.1	41.4	
		**UB**	3.6	21.9		182.5			150	19		96.9	41.4	
	**Max Conc**	**UB**	3.6	122		2570.0			150	19		196	41.4	
**Rice**	**N**					14						1		
	**Mean Conc**	**LB**				0.0						76		
		**UB**				8.9						76		
	**Max Conc**	**UB**				60.0						76		
**Rye**	**N**		1	1		18								
	**Mean Conc**	**LB**	1.8	0		11.9								
		**UB**	1.8	1		15.0								
	**Max Conc**	**UB**	1.8	1		90.0								
**Wheat**	**N**		2	2		116	1		1			41		
	**Mean Conc**	**LB**	4.8	0.3		7.7	15		38			15.6		
		**UB**	4.8	0.8		15.8	15		38			21.9		
	**Max Conc**	**UB**	9.2	1		123.0	15		38			135		

LB: lower-bound scenario where the concentration of non-detected analyte is zero and the concentration of detected but non-quantified analyte is the limit of detection. UB: upper-bound scenario where the concentration of non-detected analyte is the limit of detection and the concentration of detected but non-quantified analyte is the limit of quantification. Max Conc refers to maximum upper bound concentration value.

Table S6. Occurrence and co-occurrence of NIV, NIV3G and OTA (µg/kg) for Barley, Cereals, Maize, Oat, Rice, Rye and Wheat for feed and food products.

			FOOD			FEED		
			**NIV**	**NIV3G**	**OTA**	**NIV**	**NIV3G**	**OTA**
**Barley**	**N**		16	1	6			5
	**Mean Conc**	**LB**	35.2	25.2	1.0			10.0
		**UB**	40.2	25.2	1.1			12.0
	**Max Conc**	**UB**	180.0	25.2	5.6			25.7
**Cereals**	**N**		16		22			
	**Mean Conc**	**LB**	3.3		0.4			
		**UB**	5.5		0.4			
	**Max Conc**	**UB**	35.8		2.2			
**Maize**	**N**		21		32	89		68
	**Mean Conc**	**LB**	9.3		0.3	190.6		2.2
		**UB**	28.3		0.6	210.0		2.7
	**Max Conc**	**UB**	175.7		4.8	2547.0		51.0
**Oat**	**N**		20		4	3	1	1
	**Mean Conc**	**LB**	81.4		0.1	263.3	36.9	0.0
		**UB**	86.3		0.5	280.0	36.9	10.0
	**Max Conc**	**UB**	208.0		1.0	635.0	36.9	10.0
**Rice**	**N**		5		44			
	**Mean Conc**	**LB**	0.0		2.0			
		**UB**	16.0		2.0			
	**Max Conc**	**UB**	75.0		27.3			

**Rye**	**N**		9		5			4
	**Mean Conc**	**LB**	12.0		0.8			6.5
		**UB**	14.6		0.9			6.5
	**Max Conc**	**UB**	56.9		2.1			14.5
**Wheat**	**N**		47	1	50	19		24
	**Mean Conc**	**LB**	54.8	23.1	0.5	58.2		12.7
		**UB**	75.2	23.1	0.8	79.2		13.4
	**Max Conc**	**UB**	302.4	23.1	3.9	690.0		267.0

LB: lower-bound scenario where the concentration of non-detected analyte is zero and the concentration of detected but non-quantified analyte is the limit of detection. UB: upper-bound scenario where the concentration of non-detected analyte is the limit of detection and the concentration of detected but non-quantified analyte is the limit of quantification. Max Conc refers to maximum upper bound concentration value.

Table S7. Equivalent mycotoxin (parent and modified) concentration in food (cereal-food based products) at country level in Europe.

	AFeq (µg/kg)			DONeq (µg/kg)			FBeq (µg/kg)			NIVeq (µg/kg)			OTAeq (µg/kg)			T2+HT2eq (µg/kg)			ZENeq (µg/kg)		
	**LB**	**MEAN**	**UB**	**LB**	**MEAN**	**UB**	**LB**	**MEAN**	**UB**	**LB**	**MEAN**	**UB**	**LB**	**MEAN**	**UB**	**LB**	**MEAN**	**UB**	**LB**	**MEAN**	**UB**
**AT**	0.2	0.3	0.3	130.6	202.1	293.6															
**BA**																38.3	42.1	46.0			
**BE**				84.0	85.3	87.4										15.7	17.6	24.5	169.6	220.1	275.5
**CZ**				33.6	33.7	33.8				13.8	13.8	13.8				35.7	35.7	35.7	1.2	1.3	1.4
**DE**	0.1	0.3	0.4	207.2	207.4	207.6	126.0	126.0	126.0	178.8	190.5	202.2				28.3	28.3	28.3	24.4	24.4	24.4
**DK**				78.4	78.4	78.4	19.4	19.8	20.2	14.3	14.3	14.3				22.2	22.2	22.2	1.0	1.0	1.0
**ES**	7.3	7.6	8.0	62.2	65.7	69.4	96.5	97.6	98.7	6.6	12.1	17.7	2.2	2.2	2.3	2.6	8.1	14.2	2.2	5.3	8.5
**FI**				241.7	242.0	243.0				48.5	48.5	48.5				31.2	31.6	32.6	5.4	5.4	5.4
**FR**	0.1	0.1	0.2	23.7	24.6	25.4	20.2	20.2	20.2	2.2	2.8	3.3	0.0	0.0	0.0	0.8	1.2	1.6	1.5	1.5	1.5
**GB**	1.1	1.1	1.1	83.3	83.4	86.3	0.0	2.5	5.0	76.1	76.1	76.4				204.7	204.8	205.2	21.9	22.6	23.2
**HR**																31.8	35.6	39.5			
**IE**																53.5	53.5	53.5			
**IT**	0.5	0.5	0.5	24.7	25.6	26.5	193.0	193.0	193.0	36.7	36.7	36.7	0.1	0.2	0.2	35.4	36.2	37.0	1.3	2.2	3.0
**LT**																					
**LV**				145.5	145.5	145.5										10.7	10.7	10.7	7.1	7.1	7.1
**MK**				171.4	214.3	257.1															
**PL**	0.0	0.0	0.0	40.4	41.2	42.7	183.3	185.7	190.0	34.2	36.3	38.3	0.4	0.4	0.4	11.1	11.8	12.6	2.0	2.0	2.1
**PT**	0.0	0.1	0.3	3.8	26.3	52.7	0.0	6.3	12.5	9.4	15.7	31.3	0.2	0.2	0.3	0.0	0.4	0.8	8.8	11.4	14.0
**RO**				82.5	112.2	141.9	58.4	58.4	58.4	0.0	75.0	150.0				2.6	23.4	44.2	87.0	87.9	88.9
**RS**	0.0	0.3	0.6	42.8	43.7	44.6	0.0	0.0	0.0	0.0	2.5	5.0	0.0	1.1	2.1	6.4	7.2	8.0	0.7	1.1	1.5
**SE**	5.1	5.1	5.1	1155.0	1155.0	1155.0				123.0	123.0	123.0				50.0	50.0	50.0			

**SI**	0.0	0.2	0.4	377.7	380.0	382.3	1169.0	1204.9	1240.6				0.6	0.8	1.0	7.3	18.4	29.6	264.7	264.7	264.7
**TR**	0.5	0.8	1.1				326.7	332.9	339.2				1.2	1.2	1.3						

Countries are reported in ISO Alpha2-CODE. For each mycotoxin Lower Bound (LB), Mean and Upper Bound (UB) concentrations are reported. Within the modified mycotoxins a Relative Potency Factor was applied accordingly to [1-3]

1. EFSA. Risks for animal health related to the presence of zearalenone and its modified forms in feed. *EFSA Journal* **2017**, *15*, 4851, doi:10.2903/j.efsa.2017.4851.
2. EFSA. Risks to human and animal health related to the presence of deoxynivalenol and its acetylated and modified forms in food and feed. *EFSA Journal* **2017**, *5*, 4718, doi:10.2903/j.efsa.2017.4718.
3. EFSA. Risks for animal health related to the presence of fumonisins, their modified forms and hidden forms in feed. *EFSA Journal* **2018**, *16*, 5242, doi:10.2903/j.efsa.2018.5242.
